# Cytogenetic and molecular analysis of the holocentric chromosomes of the potato aphid *Macrosiphum euphorbiae* (Thomas, 1878)

**DOI:** 10.3897/CompCytogen.v5i3.1724

**Published:** 2011-08-24

**Authors:** Valentina Monti, Gian Carlo Manicardi, Mauro Mandrioli

**Affiliations:** 1 Dipartimento di Biologia, Università di Modena e Reggio Emilia, Via Campi 213/D, 41125 Modena, Italy; 2 Dipartimento di Scienze Agrarie e degli Alimenti, Università di Modena e Reggio Emilia, Via Amendola 2, 42100 Reggio Emilia, Italy

**Keywords:** aphid, holocentric chromosomes, telomeres, heterochromatin, NOR heteromorphism

## Abstract

Cytogenetic and molecular investigations on the holocentric chromosomes of the aphid *Macrosiphum euphorbiae* (Thomas, 1878)have been carried out using silver staining and C-banding (followed by chromomycin A3 and DAPI staining) in order to improve our knowledge about the structure of aphid chromosomes. The C-banding pattern is peculiar since only the two X chromosomes and a single pair of autosomes presented heterochromatic bands. Silver staining and FISH with the 28S rDNA probe localized the rDNA genes on one telomere of each X chromosome that were also brightly fluorescent after chromomycin A3 staining of C-banded chromosomes, whereas all other heterochromatic bands were DAPI positive. Interestingly, a remarkable nucleolar organizing region (NOR) heteromorphism was present making the two X chromosomes easily distinguishable. Southern blotting and FISH assessed the presence of the (TTAGG)*n* repeat at the ends of all the *Macrosiphum euphorbiae* chromosomes. Karyotype analysis showed that all males possessed the X chromosome with the larger amount of rDNA suggesting a non-Mendelian inheritance of the two X chromosomes.

## Introduction

In the last decades classic and molecular cytogenetics provided an integrated approach for the structural, functional and evolutionary analysis of aphid holocentric chromosomes (Blackman 1980, Manicardi et al. 1998, Mandrioli et al. 1999a, 1999b, Manicardi et al. 2002, Mandrioli et al. 2011).

Interest in aphid cytogenetics is mostly due to the holocentric/holokinetic structure of their chromosomes that present a diffused centromeric activity (Wrensch et al. 1994). This particular type of chromatin organization has been described in almost all the eukaryotic taxa examined so far, with the exception of echinoderms and chordates (Wrensch et al. 1994).

Aphids, in view of the ease with which mitotic chromosome can be obtained from embryonic tissues, represent an ideal model to better understand the architecture of holocentric chromosomes, and to work out the differences/similarities with monocentric ones (Manicardi et al. 2002). At the same time, a cytogenetic analysis of aphids is very helpful since the description of species-specific chromosomal markers could make easier the identification of species that is, at present, quite difficult (Rakauskas 1998, Blackman 1980).

Aphid X chromosomes have been studied with great attention since they present several structural constraints (Manicardi et al. 2002). In particular, the X chromosomes showed a large amount of heterochromatin and possessed the rDNA cluster located at one telomere in almost all the species studied at a cytogenetic level (Mandrioli et al. 1999a, b, Manicardi et al. 2002). Furthermore, aphid X chromosomes seem to be more stable than autosomes since, when fragmentations occur, they mostly affected autosomes leaving X chromosomes usually as the longest in aphid karyotype (Khuda-Buksh and Datta 1981, Blackman 1987, Hales 1989).

In order to better understand X chromosome evolution in aphids, we decided to carry out a cytogenetic analysis of the holocentric chromosomes of the aphid *Macrosiphum euphorbiae* (Thomas, 1878), an important pest of several crops, belonging to a genus that has been up to date scarcely studied at a cytogenetic level (Blackman 1980).

## Material and methods

Specimens of *Macrosiphum euphorbiae* were collected on *Bellis perennis* (Linnaeus, 1753) in Modena (Italy) and maintained at 22 C with 16:8 hours light/darkness on *Bellis perennis* plants. Male aphids were obtained by exposing parthenogenetic females to short photoperiods (8:16 hours light/darkness) according to Crema (1979).

Chromosome preparations from 150 parthenogenetic females were made by spreading embryo cells, as described by Manicardi et al. (1996). Male chromosomes have been obtained by squash preparation of 30 embryos as reported by Manicardi et al. (1991).

C banding treatment was performed according to the technique of Sumner (1972). C banded chromosomes were stained with DAPI according to Donlon and Magenis (1983) and with chromomycin A3 (CMA3) as described in Schweizer (1976). NOR regions were labelled by silver staining following the technique of Howell and Black (1980).

DNA extraction from aphid embryos was performed as described in Mandrioli et al. (1999a).

The 28S rDNA probe was obtained by PCR amplification of a 400 bp long fragment of the 28S rDNA gene using the two primers, F (5’-AACAAACAACCGATACGTTCCG) and R (5’-CTCTGTCCGTTTACAACCGAGC), designed according to the insect 28S rRNA sequences available in GenBank. The amplification mix contained 100 ng genomic DNA, 1 mM of each primer, 200 mM dNTPs and 2 U of DyNAZyme II polymerase (Finnzymes Oy). Amplification was performed using a Hybaid thermal-cycler at an annealing temperature of 60°C for 1 min with an extension time of 1 min at 72°C.

In order to test the presence of the telomeric (TTAGG)*n* repeat, a probe was obtained by PCR amplification using the two primers F (TTAGG)5 and R (CCTAA)5 in the absence of template, as described by Ijdo et al. (1991).

The telomeric and 28S rDNA probes were labelled using the PCR DIG labelling mix (Roche) according to the Roche protocols.

Southern blotting and fluorescent *in situ* hybridization (FISH) were made as described by Mandrioli et al. (1999a). FISH slides were observed using a Zeiss Axioplan epifluorescence microscope equipped with a 100 W mercury light source. Photographs of the fluorescent images were taken using a CCD camera (Spot, Digital Instrument, Madison, USA) and using the Spot software supplied with the camera and processed using Adobe Photoshop (Adobe Systems, Mountain View, CA).

## Results

**Figure 1a–h.**
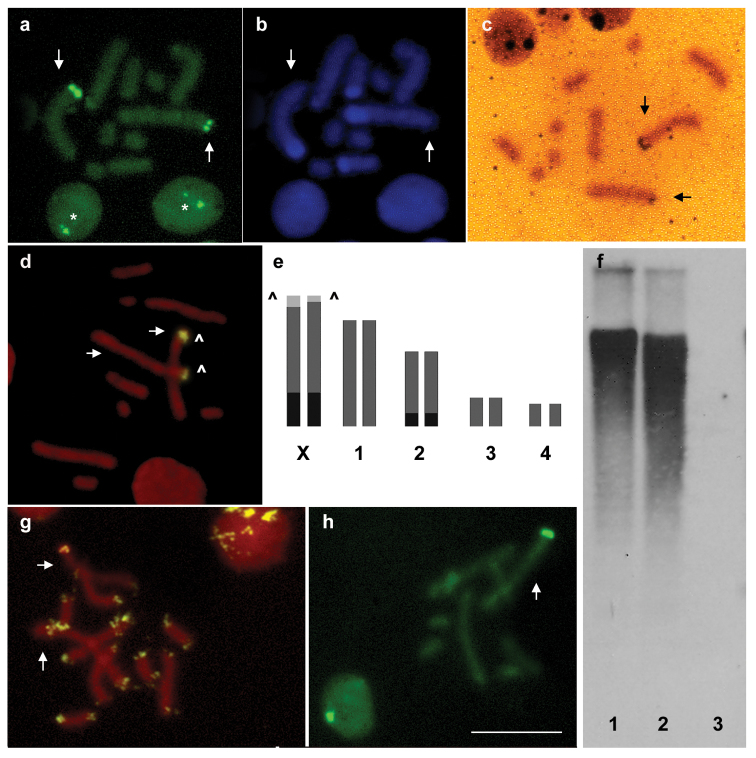
*Macrosiphum euphorbiae* chromosomes, stained with CMA3 **(a)** and DAPI **(b)** after C banding, showing heterochromatin on one telomere of the two X chromosomes and on autosome pair 2. Silver staining **(c)** and FISH with a 28S rDNA probe **(d)** evidenced heteromorphic NORs located at one telomere of each X chromosome that was also fluorescent after CMA3 staining, as summarized in the panel **(e)** Southern blotting **(f)** after digestion with *Xho*I of DNA samples of *Acyrthosiphon pisum* **1** *Macrosiphum euphorbiae* **2** and *Drosophila melanogaster* **3** together with FISH **(g)** assessed that the telomeric sequence (TTAGG)*n* constitute each chromosomal end of the *Macrosiphum euphorbiae* chromosomes. CMA3 staining of male chromosomes showed that all the male plates present the X chromosome with the larger NOR **(h)** Arrows indicate X chromosomes. Arrowheads indicate NORs. Asterisks evidence the presence of heteromorphic nucleoli in the *Macrosiphum euphorbiae* nuclei. Bar = 10 µm.

The parthenogenetic females of *Macrosiphum euphorbiae* showed a chromosome number of 2n=10 (Fig. 1). C banding followed by CMA3 staining showed a bright fluorescence exclusively limited to one telomere of the two longest chromosomes (Fig. 1a) that, on the basis of the comparison with male karyotype, have been identified as X chromosomes. DAPI staining showed a large heterochromatic band at the opposite end of the X chromosomes in respect to the GC-rich CMA3-stained telomere. A second heterochromatic band was observed at one telomere of the autosome pair 2 (Fig. 1b).

The overlapping between CMA3 areas and rDNA genes has been confirmed by both silver staining (Fig. 1c) and FISH with the 28S rDNA probe (Fig. 1d) showing an exclusive localization of the rDNA genes on *Macrosiphum euphorbiae* X chromosomes. Silver staining, FISH and CMA3 staining demonstrated the remarkable occurrence of heteromorphism between homologous NORs on X chromosomes (Figs. 1a, c, d) allowing us to distinguish the two X chromosomes. The same heteromorphism was also evident in interphase nuclei (Fig. 1a).

The presence of the (TTAGG)*n* repeat has been evaluated by Southern blotting and FISH. Southern blotting revealed a diffuse smear of hybridization (Fig. 1f), whereas FISH experiments with the telomeric (TTAGG)*n* probe showed bright FITC-fluorescent spots at the ends of all chromosomes (Fig. 1g). In the interphase nuclei of *Macrosiphum euphorbiae*, telomeres clustered in few highly fluorescent foci (Fig. 1g).

The male karyotype consisted of 8 autosomes and one X chromosome only (Fig. 1h). Interestingly, all the analysed males possessed the X chromosome with the larger NOR.

## Discussion

Currently, more than 4000 aphid species have been described, but the chromosome number has been reported only for about 500 of them (Blackman 1980). Furthermore, less than 10% of the described aphid karyotypes have been deeply studied at a cytogenetic level, even if aphids are intriguing model in animal cytogenetics in view of the holocentric nature of their chromosomes (Manicardi et al. 2002).

According to our results, *Macrosiphum euphorbiae* has a chromosome number of 2n=10, which represents the typical diploid chromosome number reported in the literature for species of this genus (Blackman 1980).

C banding carried out on *Macrosiphum euphorbiae* mitotic chromosomes revealed that heterochromatin was not equilocated on each chromosome, but limited to telomeric regions of the two X chromosomes and to autosome pair 2. A preferential storage of heterochromatin on X chromosomes has been previously observed in almost all the aphid species cytogenetically studied to date with the exception of *Diuraphis noxia* (Mordvilkoex Kurdjumov, 1913) (Novotna et al. 2011), but C-bands on a single autosome pair were reported only in *Acyrthosiphon pisum* (Harris, 1776) (Bizzaro et al. 2000) and *Brevicoryne brassicae* (Linnaeus, 1758) (Giannini et al. 2003).

The different responses to CMA3 and DAPI staining after C banding point out a DNA composition heterogeneity of *Macrosiphum euphorbiae* heterochromatin. Indeed, GCrich NOR-associated heterochromatin differs from all other heterochromatic bands that are made by AT-rich DNAs. This pattern of heterochromatin heterogeneity seems to be a general characteristic of aphid chromatin since it has been described in all species investigated so far at a cytogenetic level (Manicardi et al. 2002).

Contrary to the protocol followed for several aphid species, in order to induce a clear-cut banding pattern on *Macrosiphum euphorbiae* chromosomes, we modified the usual C-banding procedure making a 7 minutes long barium hydroxide treatment. Difficulties in obtaining clear-cut C-banding have been recently reported in the aphid *Diuraphis noxia* (Novotna et al. 2011). Interestingly, both these species belong to the tribe Macrosiphini, whereas most of the species with a clear cut banding were Aphidini. Further studies on the tribe Macrosiphini could shed light on the peculiarities of their chromatin organization, which are at the basis of this different banding propensity.

Southern blot experiments with the (TTAGG)*n* probe showed smears in *Macrosiphum euphorbiae* genome suggesting that its telomeresare composed ofTTAGG repeats. This result has been confirmed by FISH experiments that clearly showed a hybridization signal on each telomere, whereas no evidence of any interstitial labelling has been observed demonstrating that the TTAGG repeats are restricted to the terminal regions of all aphid chromosomes. The presence of the (TTAGG)*n* repeat in *Macrosiphum euphorbiae* further support the hypothesis that this telomeric sequence is common in aphids (Bizzaro et al. 2000, Monti et al. 2011), so that its absence in *Diuraphis noxia* (Novotna et al. 2011) could be considered an exception, as previously reported in other insects (Frydrychovà et al. 2004, Lukhtanov and Kuznetsova 2010).

In interphase nuclei of most organisms the telomeric regions are situated in an ordered fashion with an association to the nuclear matrix and clustering at least in some stage of cell life (Palladino et al. 1993, Luderus et al. 1996, Pryde et al. 1997). Accordingly to previous results in aphids (Monti et al. 2011), telomeres appeared clustered into few foci in the *Macrosiphum euphorbiae* nuclei and were not located mainly near the nuclear periphery as reported in other insects such as *Drosophila melanogaster* (Meigen, 1830) (Hochstrasser et al. 1986) and the cabbage moth *Mamestra brassicae* (Linnaeus, 1758) (Mandrioli 2002).

NOR number and position have been frequently reported as highly variable in insects, where rDNA genes have been frequently found also on autosomes or only on autosomes as generally reported in Lepidoptera and Psylloidea (Maryańska-Nadachowska et al. 1992, Maryańska-Nadachowska and Grozeva 2001, Nguyen et al. 2010), including several species with multiple NORs (e.g. Postiglioni and Brum-Zorrilla 1988, Juan et al. 1993). Silver staining of *Macrosiphum euphorbiae* mitotic metaphases revealed two dots located on one telomere of each X chromosome. This seems to be a highly conservative characteristic of aphid chromosomes, since the same NOR localization has been described in almost all the aphids species studied to date (Kuznetsova and Gandrabur 1991, Kuznetsova and Maryańska-Nadachowska 1993, Blackman and Spence 1996, Manicardi et al. 1998, 2002), with the unique exception of *Schoutedenia ralumensis* (Rübsaamen, 1905) and *Maculolachnus submacula* (Walker, 1848) that present autosomal NORs and *Amphorophora idaei* (Borner, 1839) showing interstitial NORs on X chromosomes (Blackman 1987).

In several aphid species silver staining revealed the occurrence of an appreciable level of heteromorphism between homologous NORs due to a different distribution of rDNA genes between the two X chromosomes, but, in all the previous studies, different levels of heteromorphism have been observed both at inter- and intra-individual levels (Blackman and Spence 1996, Mandrioli et al. 1999b; Manicardi et al. 1998, 2002). Contrarily to previous observations, all the observed *Macrosiphum euphorbiae* plates presented an X chromosome with a NOR region larger than the homologue allowing us to clearly differentiate the two sex chromosomes. In view of this stable chromosomal marker we performed experiments of male determination in order to evaluate if during this process both the X chromosomes have the same chances of being inherited in males.

All the parthenogenetic eggs during the prophase present two X chromosomes linked by NORs (Schrader 1940, Orlando 1974, Hales and Mitler 1983, Blackman and Hales 1986). However, in eggs developing as females, the connection is quickly lost, but in male generating eggs the X chromosomes remain attached by sticky NORs and undergo a sort of non-canonical reductional division (Blackman and Hales 1986). At the end of this peculiar division the egg has one X chromosome only and it is determined as a male.

The observation that all the *Macrosiphum euphorbiae* male metaphases had an X chromosome with a large NOR evidenced a selective bias favouring X chromosomes with larger number of rDNA genes. This fact could be due by a non-random elimination of one X chromosome during male determination process or by the early abortion of embryos containing an X chromosome with few rDNA genes. Contrarily to what has been observed in *Megoura viciae* (Buckton, 1876) (Crema 1981), we never observed the presence of aborted eggs among the developing male embryos of *Macrosiphum euphorbiae* so that we hypothesized that the bias occurred during the male determination process which ended with a selective elimination of the X chromosome bearing few rDNA genes.

The phenomenon of biased inheritance of X chromosomes in aphids seems to be controversial among aphids since observations on *Sitobion fragariae* (Walker, 1848), using an X-linked polymorphic microsatellite marker, suggested that X chromosome loss during male determination was random (Wilson et al. 1997), whereas the presence of strong biases in the transmission of sex chromosomes has been reported in *Rhopalosiphum padi* (Linnaeus, 1758) (Franz et al., 2005)

On the basis of our results on *Macrosiphum euphorbiae*, we suggest that the presence of an unequal distribution of rDNA between the two X chromosomes could affect the attachment of the X chromosome by sticky NORs favouring the loss of the X chromosome with few rDNA genes and a not random inheritance of the X chromosomes. On the contrary, aphid specimens with an equal distribution of rDNA genes could undergo a random loss of the X chromosomes during male determination. On the basis of our hypothesis, therefore, NOR heteromorphism does not inhibit male determination (as previously reported by Blackman and Spence 1996), but affects the inheritance of the X chromosomes making it non random.
